# P‐Coumaric Acid Improves Skeletal Muscle Atrophy in Chronic Kidney Disease by Modulating TLR4/MyD88/NF‐κB‐Mediated Inflammation and Oxidative Stress

**DOI:** 10.1111/jcmm.70659

**Published:** 2025-07-17

**Authors:** Hao Wang, Chi Zhang, Jinyue He, Zhuoen He, Shihua Yan, Yuan Zhang, Shiyin Huang, Yangtian Yan, Yuchi Chen, Zhiqiang Xian, Rong Hu, Yanjing Wang, Wei Xiao, Mingqing Wang

**Affiliations:** ^1^ School of Traditional Chinese Medicine Southern Medical University Guangzhou Guangdong China; ^2^ Department of Traditional Chinese Medicine, Nanfang Hospital Southern Medical University Guangzhou Guangdong China; ^3^ Department of Traditional Chinese Medicine, Zhujiang Hospital Southern Medical University Guangzhou Guangdong China; ^4^ Key Laboratory of Glucolipid Metabolic Disorder, Ministry of Education Guangdong Pharmaceutical University Guangzhou Guangdong China; ^5^ TCM‐Integrated Hospital of Southern Medical University Guangzhou Guangdong China

**Keywords:** inflammation, oxidative stress, P‐Coumaric acid, skeletal muscle atrophy, TLR4/MyD88/NF‐κB pathway

## Abstract

Skeletal muscle atrophy is a prevalent complication in chronic kidney disease (CKD), and its pathogenesis is closely related to inflammation and oxidative stress. P‐Coumaric acid (PCA) is a phenolic acid with anti‐inflammatory and antioxidant pharmacological actions. This research aims to investigate the effect of PCA on CKD‐induced muscle atrophy and its underlying mechanism. In our study, in vivo and in vitro models were established by using 5/6 nephrectomized rats and LPS‐induced C2C12 myoblasts. The experimental results showed that PCA ameliorated kidney injury in CKD rats and increased skeletal muscle weight and the cross‐sectional area of muscle fibres. In both CKD rats and LPS‐induced C2C12 myoblasts, PCA also exhibited anti‐inflammatory and antioxidant effects, reduced the levels of pro‐inflammatory cytokines and enhanced the activity of antioxidant enzymes. Network pharmacology studies have identified 165 common targets between PCA and skeletal muscle atrophy. Furthermore, the experimental results also demonstrated that PCA decreased the expression of TLR4, MyD88, NF‐κB p65, MurF1 and MAFbx at both the protein and mRNA levels. Additionally, in vitro experiments showed that the use of TLR4 agonists could reverse the muscle‐protective effect of PCA. In summary, this study illustrated that PCA ameliorated skeletal muscle atrophy in CKD rats by inhibiting the TLR4/MyD88/NF‐κB pathway.

**Abbreviations**
Alb: albumin
AUC: area under the curve
BUN: blood urea nitrogen
CA: cinnamic acid
CAT: catalase
CKD: chronic kidney disease
CRP: C‐reactive protein
CSA: cross‐sectional area
EAT: epididymal adipose tissue
GA: gastrocnemius
GSH‐Px: glutathione peroxidase
H&E: haematoxylin–eosin
IKKβ: inhibitor kappa B kinase β
IPGTT: intraperitoneal glucose tolerance test
LPS: lipopolysaccharide
MAFbx: muscle atrophy F‐box
MDA: malondialdehyde
MurF1: muscle‐specific ring finger 1
MyD88: myeloid differentiation factor 88
NF‐κB: nuclear factor kappa B
PA: palmitic acid
PCA: p‐Coumaric acid
PEW: protein‐energy wasting
PPI: protein–protein interaction
qRT‐PCR: Quantitative real‐time polymerase chain reaction
ROS: reactive oxygen species
Scr: serum creatinine
SMA: skeletal muscle atrophy
SOD: superoxide dismutase
TA: tibialis anterior
TEM: transmission electron microscopy
TLR4: toll‐like receptor 4
TNF‐α: tumour necrosis factor‐alpha

## 1 Introduction

Protein‐energy wasting (PEW) is a prevalent complication and the main cause of increased morbidity and mortality in CKD patients [1, 2, 3]. Skeletal muscle wasting is an important clinical manifestation of PEW and a reliable diagnostic indicator [4]. In some cases, it is even considered synonymous with PEW [5]. Moreover, increased muscle atrophy correlates with a threefold increase in mortality risk within 4 to 6 years [6], and addressing this atrophy can treat and slow the progression of CKD‐PEW and lower the risk of death [7].

Studies indicate that skeletal muscle atrophy in CKD stems from multiple causes, and various mediators disrupt the equilibrium between protein synthesis and decomposition, leading to a sustained metabolic imbalance and the eventual onset of muscle wasting [5, 8]. The typical characteristics of chronic muscle inflammation are manifested by elevated levels of pro‐inflammatory cytokines, increased muscle protein breakdown, reduced muscle mass and strength and shifts in muscle fibre types [9, 10]. Pro‐inflammatory cytokines such as TNF‐α directly act on muscle cells, inhibiting muscle protein synthesis and promoting its degradation, resulting in skeletal muscle wasting [11, 12]. Oxidative stress is an internal defence mechanism, but it represents an unbalanced state between excessive oxidation and the antioxidant system [13]. Existing studies have emphasized the role of excessive reactive oxygen species (ROS) and oxidative stress in skeletal muscle atrophy [14]. It has been shown that increased oxidative stress may increase mitochondrial dysfunction and activate the autophagy process, leading to excessive accumulation of ROS and exacerbation of muscle inflammation [15]. The augmented inflammatory response increases the generation and liberation of ROS, while oxidative stress accelerates the triggering of inflammatory signalling pathways and the release of inflammatory factors [16, 17]. The degradation of muscle proteins caused by both of them undermines muscle function, rendering it more vulnerable to the impacts of oxidative stress and inflammation, thereby continuously worsening the extent of skeletal muscle atrophy and giving rise to a vicious cycle. These manifestations highlight that inflammation and oxidative stress are two pivotal and interconnected molecular mechanisms within the pathogenesis of skeletal muscle atrophy [18].

P Coumaric acid (PCA), also known as 4‐hydroxycinnamic acid, is a phenolic compound containing a phenolic hydroxyl group, abundantly present in traditional Chinese medicines such as *Panax ginseng C. A. Mey* [19, 20], *Sparganii Rhizoma* [21], *Phragmitis rhizoma* [22] and *Tradescantia spathacea* [23], as well as in diverse plant‐derived foods such as fruits, vegetables, tea, coffee and cereals [24]. Pharmacological studies indicate that PCA possesses antioxidant, anti‐inflammatory, anti‐tumour and neuroprotective properties [25, 26]. Studies on structure–activity relationships have shown that the antioxidant activity of hydroxycinnamic acids is related to the number and position of phenolic hydroxyl groups [27, 28]. The phenolic hydroxyl group in PCA can provide hydrogen atoms to scavenge ROS, terminate the free radical chain reactions of ROS and facilitate electron transfer through conjugation with the benzene ring and double bond side chain to form stable products, thereby exerting antioxidant effects [29]. Therefore, the antioxidant capacity is the foundation of PCA's diverse pharmacological benefits [25]. PCA can help guard against diseases closely related to oxidative stress damage, such as inflammation, cancer and hyperlipidaemia, and help improve the body's resistance to chronic or acute injurious diseases [30]. Recent experiments have demonstrated that PCA has a therapeutic impact on diseases such as myocardial infarction and liver fibrosis through its anti‐inflammatory and antioxidant properties [31, 32]. However, PCA has rarely been explored in the context of skeletal muscle atrophy.

Based on these findings, it was hypothesized that PCA could potentially ameliorate CKD‐induced skeletal muscle atrophy through its anti‐inflammatory and antioxidant functions. To validate this hypothesis, we conducted in vivo evaluations of skeletal muscle weight and the cross‐sectional area of muscle fibres in CKD rats to examine the protective effects of PCA on skeletal muscles. We also measured inflammatory factors and oxidative stress markers in the muscles and serum of CKD rats, as well as in LPS‐induced C2C12 myoblasts, to elucidate the anti‐inflammatory and antioxidant properties of PCA. Moreover, network pharmacology and molecular docking techniques were employed to predict the molecular mechanisms through which PCA improves skeletal muscle atrophy, with special attention paid to the inflammatory signalling pathway mediated by TLR4. Ultimately, our study demonstrated that PCA might enhance its anti‐inflammatory and antioxidant capacities and reduce protein degradation by inhibiting the TLR4/MyD88/NF‐κB signalling pathway, thereby alleviating muscle wasting in CKD rats. These results suggest that PCA may hold promise as a potential therapeutic agent for treating CKD‐induced skeletal muscle atrophy and other inflammation‐ and oxidative stress‐related diseases.

## 2 Materials and Methods

### 2.1 Materials and Reagents

PCA (molecular formula: C9H8O3; Purity > 98%, C108514) was obtained from Aladdin Biochemical Technology Co. Ltd. (Shanghai, China). Assay kits for serum creatinine (Scr, C011‐2‐1), blood urea nitrogen (BUN, C013‐2‐1), urinary protein (C035‐2‐1), serum albumin (Alb, A028‐2‐1), catalase (CAT, A007‐1‐1), glutathione peroxidase (GSH‐Px, A005‐1‐2) and malondialdehyde (MDA, A003‐1‐2) were purchased from Jiancheng Bioengineering Institute (Nanjing, China). The superoxide dismutase assay kit (SOD, S0101S‐100) was purchased from Beyotime Biotechnology Co. Ltd. (Shanghai, China). ELISA kits for leptin, TNF‐α, IL‐6 and CRP were purchased from Jingmei Biotechnology Co. Ltd. (Jiangsu, China). High‐glucose DMEM was purchased from Gibco (USA); foetal bovine serum (FBS, FSP500) was purchased from ExCell Biological Co. Ltd. (Suzhou, China); and penicillin/streptomycin was purchased from NCM Biotechnology Co. Ltd. (Suzhou, China). Lipopolysaccharide (LPS, L8274) and palmitic acid (PA, P0500) were purchased from Sigma‐Aldrich (USA). TRIZOL reagent was purchased from Thermo Fisher Scientific (USA). Rever Tra Ace qPCR RT Kit and SYBR Green Realtime PCR Master Mix were purchased from Toyobo (Japan). The DCFH‐DA fluorescent probe (C263) was purchased from GeneCopoeia (USA). Antibodies for TLR4 (66350‐1‐Ig), MyD88 (67969‐1‐Ig), MurF1 (55456‐1‐AP) and GAPDH (HRP‐60004), as well as HRP‐conjugated anti‐Mouse IgG (SA00001‐1) and HRP‐conjugated anti‐Rabbit IgG (SA00001‐2), were purchased from Proteintech (Wuhan, China). NF‐κB p65 (#4764) and p‐NF‐κB p65 (#9475) antibodies were purchased from Cell Signalling Technology (USA). MAFbx antibody (ab157596) was purchased from Abcam (UK).

### 2.2 Animal Experiment

With permission number SCXK 2021–0041, male Sprague–Dawley (SD) rats, weighing 160–180 g, were purchased from the Experimental Animal Centre of Southern Medical University (Guangzhou, China). They were housed in the animal laboratory with temperature‐controlled conditions (20°C–26°C), humidity (40%–70%) and 12‐h light/dark cycles, provided with specific pathogen‐free food and water. All animal experimental studies were subject to review and approval by the Institutional Animal Care and Use Committee of Southern Medical University (Grant No.: 2022056) and were conducted following ethical principles. After a one‐week adaptation period, 35 rats were randomized into either the 5/6 nephrectomy (Nx) group or the sham‐operation group, and the 5/6 Nx group performed a 2‐step surgical procedure to remove 5/6 of the kidneys, as previously outlined [33]. In the first step of the Nx procedure, the 2 poles (approximately 2/3) of the left kidney were excised. After 1 week of recovery, the right kidney was removed. In contrast, the kidneys of the sham‐operation group were merely exposed, unencapsulated and then placed back into the peritoneal cavity. Penicillin was injected intramuscularly (10 U per rat) 3 days after surgery to prevent infection. The body weight, motor and mental state of rats were monitored weekly after operation. Nine weeks post‐operation, Scr and BUN levels were remarkably elevated in the 5/6 Nx group relative to the sham‐operation group, indicating the successful establishment of the CKD model.

Rats that successfully completed the 5/6 Nx were split into 4 groups at random (*n* = 7): CKD, CKD + PCA 50 mg/kg (PCA‐50), CKD + PCA 75 mg/kg (PCA‐75) and CKD + PCA 100 mg/kg (PCA‐100). The sham and CKD groups were orally given a 5% carboxymethyl cellulose (CMC) suspension, while the PCA groups were administered PCA solution at doses of 50 mg/kg, 75 mg/kg and 100 mg/kg dissolved in 5% CMC suspension, respectively, once daily for 10 weeks. The dosing regimen of PCA was based on toxicological data and literature evidence [24, 34].

### 2.3 Intraperitoneal Glucose Tolerance Test (IPGTT)

After 10 weeks of PCA gavage feeding, the IPGTT was conducted to serve as an assessment of insulin resistance. After a 12‐h fasting period, the rats were intraperitoneally injected with 50% (w/v) glucose solution (2 g/kg). Blood samples were collected from the rats' tails at baseline (0 min) and at 30, 60, 90 and 120 min after glucose injection to measure blood glucose levels.

### 2.4 Specimen Collection

After completing 10 weeks of PCA gavage, 24‐h urine samples were collected from each rat. When the last dosage was given, the rats were anaesthetized, and a V‐shaped incision was made in the abdominal skin to access the abdominal aorta for blood collection via a tube. Subsequently, the rats were euthanized, with their hind limbs amputated to separate the tibialis anterior (TA), gastrocnemius (GA) and soleus muscles, which were then weighed individually and stored at −80°C. Additionally, epididymal adipose tissue (EAT) was isolated and weighed, while the kidneys were collected and stored at −80°C.

### 2.5 Detection of Renal Function and Nutritional Status Indicators

The collected blood was centrifuged at 4°C and 3000 rpm for 15 min to obtain serum. Renal function indicators, including Scr, BUN and 24‐h urinary protein, as well as the nutritional status indicator Alb, were detected according to protocols provided with the respective assay kits.

### 2.6 Haematoxylin–Eosin (H&E) Staining

Following the sacrifice, the left TA, GA, soleus muscles and kidneys of the rats were excised and preserved in 4% paraformaldehyde for one day. Subsequently, these tissues (the left TA, GA, soleus muscles and kidneys) underwent dehydration, paraffin embedding and sectioning into 4–5 μm slices. The sections were dewaxed in xylene, rehydrated in a gradient series of alcohols, stained with haematoxylin and eosin, differentiated in hydrochloric acid‐ethanol solution, washed in running water for 10 min, dehydrated and finally sealed with neutral resin. The pathological status of the kidneys and the cross‐section of skeletal muscle fibres were observed using a light microscope. Using ImageJ software (USA), the cross‐sectional area (CSA) of muscle fibres was measured.

### 2.7 Transmission Electron Microscopy (TEM)

Fresh GA muscle tissue was cut into 10 mm^3^ pieces and initially fixed in a 0.1 M cacodylate buffer solution containing 0.5% glutaraldehyde, 2% paraformaldehyde, 7% glucose and 4% polyvinylpyrrolidone, then rinsed with 0.1 M PBS buffer. Following this initial fixation, the samples were further fixed in 0.1 M cacodylate buffer containing 2% osmium acid for one hour. The samples then underwent dehydration, embedding and sectioning to produce slices approximately 0.1 μm in thickness. Ultimately, the sections were examined using transmission electron microscopy.

### 2.8 Detection of Inflammatory Cytokines and Oxidative Stress Markers

The concentration of leptin and the levels of the inflammatory cytokines TNF‐α, IL‐6 and CRP in rat serum were measured using ELISA kits. Oxidative stress markers of SOD, CAT, MDA and GSH‐Px in rat serum, muscle tissues and C2C12 myoblasts were assessed with corresponding detection kits. The experiments were conducted using the isolated serum described above. All procedures were performed in line with the manufacturer's directions for each kit.

### 2.9 qRT‐PCR Analysis

Total RNA was extracted from rat muscle tissues and C2C12 myoblasts using TRIZOL and then reverse‐transcribed into cDNA using the Rever Tra Ace qPCR RT Kit according to the manufacturer's instructions. Subsequently, qRT‐PCR was conducted with the following components: 5 μL of cDNA, 0.5 μL each of forward and reverse primers and 6 μL of SYBR Green Master Mix. The thermal cycling profile included an initial denaturation at 95°C for 600 s, followed by 40 cycles of denaturation at 95°C for 15 s and annealing/extension at 60°C for 60 s. Species‐specific primers are listed in Table S1. The data were analysed using the 2^−ΔΔCt^ method and normalized to GAPDH for accurate comparison.

### 2.10 Network Pharmacology and Molecular Docking

#### 2.10.1 Screening of PCA Targets

The potential targets of PCA were acquired from Swiss Target Prediction, TargetNet, Batman, TCMSP and other databases [35, 36]. The Genecards database [37] was used to acquire disease‐related targets by entering the keyword ‘skeletal muscle atrophy’. The shared targets between PCA and skeletal muscle atrophy constitute the primary focus of our study. To accomplish this, the Jvenn online tool was used to generate a Venn diagram and identify common targets.

#### 2.10.2 Protein–Protein Interaction (PPI) Network Analysis

The STRING database was utilized to analyse the protein–protein interactions among the common targets identified between PCA and skeletal muscle atrophy. It is worth mentioning that when using the STRING database, the species filter was adjusted to ‘ *Homo sapiens’* , and the interaction score threshold was established at 0.4 [38]. Subsequently, the PPI network was created by importing the protein–protein interaction data into Cytoscape 3.10.0 for visualization.

#### 2.10.3 KEGG Pathway and GO Enrichment Analysis

To explore the biological functions of the targets shared by PCA and skeletal muscle atrophy, the UniProt IDs of these targets were input into the DAVID database, with human specified as the species source. The criteria were set to *p*‐value < 0.01 and Corrected *p*‐value < 0.01 to acquire data for GO enrichment and KEGG pathway analysis. Finally, the WeGene online tool was employed to visualize the KEGG pathways and GO enrichment results.

#### 2.10.4 Molecular Docking Technology

According to the PPI network diagram and experimental results, the key targets TLR4, MyD88, IKKβ, NF‐κB p65 and MurF1 were selected for molecular docking verification. First, the three‐dimensional structures of PCA and its phenolic hydroxyl‐deficient derivative, cinnamic acid (CA), were acquired from the PubChem database and then optimized through energy minimization using Chem3D software. Subsequently, the structures of TLR4 (PDB ID: 2Z62), MyD88 (PDB ID: 4EO7), IKKβ (PDB ID: 3BRV), NF‐κB p65 (PDB ID:3RC0) and MurF1 (PDB ID: 4JNW) proteins were obtained from the Protein Data Bank (PDB). These structures were then subjected to dehydration and hydrogenation using AutoDock software, followed by molecular docking simulations. Finally, the docking results were visualized with PyMol software.

### 2.11 Cell Culture and Treatments

Mouse C2C12 myoblasts (primary culture cells) were sourced from the Cell Bank of the Chinese Academy of Sciences. The myoblasts were maintained in an incubator with 5% CO_2_ at 37°C and cultured in high‐glucose DMEM supplemented with 10% FBS and 100 U/mL penicillin–streptomycin solution. To test the cytotoxic effects of PCA, LPS and PA, different concentrations of PCA (0, 10, 20, 40, 60, 80, 100 μM), LPS (0, 0.1, 0.2, 0.4, 0.8 μg/mL) and PA (0, 12.5, 25, 50, 100, 200 μM) were tested on C2C12 myoblasts for 24 h. Based on the cell viability results, 0.4 μg/mL was selected as the optimal concentration for LPS‐induced inflammation and oxidative stress in C2C12 myoblasts. LPS (0.4 μg/mL) was combined with different concentrations of PCA (10, 20, 40, 60 μM) and incubated for 24 h to determine the most effective therapeutic concentration of PCA in vitro. The viability of the cells was assayed through the utilization of the MTT method. Finally, C2C12 myoblasts were categorized into 4 groups: control, LPS, LPS + PCA and LPS + PCA + PA, to elucidate the mechanism of action of PCA.

### 2.12 MTT Assay

C2C12 myoblasts were inoculated into 96‐well plates (5000/well) and processed in the manner outlined above. After incubation for 24 h at 37°C in a 5% CO_2_ environment, the culture medium was substituted with an MTT solution (0.5 mg/mL) in a DMEM medium. After further incubation for 4 h, the MTT solution was discarded, and 100 μL dimethyl sulfoxide (DMSO) was added to each well and shaken gently in the dark for 15 min. Then, the absorbance at 570 nm was determined with a microplate reader. Finally, the cell viability of each group was calculated and compared.

### 2.13 Cellular ROS Measurements

C2C12 myoblasts were inoculated into 96‐well plates (5000/well) and subsequently processed in accordance with the aforementioned methodology. After 24 h of culture, the cells were incubated with 2,7‐dichlorofluorescein diacetate (H2DCFDA) solution (10 μM, 100 μL/well) for 30 min at 37°C in a 5% CO_2_ incubator in the dark. After that, each well was washed twice with PBS, and the fluorescence intensity of each well was determined using a fluorescent microplate reader, with measurements taken at excitation and emission wavelengths of 485 nm and 530 nm, respectively. The intracellular ROS levels were normalized to the blank control and used to present the results. Additionally, four typical photos were arbitrarily captured from each group under a fluorescence microscope.

### 2.14 Western Blot Analysis

Proteins were extracted from rat muscle tissue and C2C12 myoblasts using RIPA lysis buffer, and protein concentrations were quantified. Protein samples were subsequently separated by 10% SDS‐PAGE and transferred at 300 mA for 90 min to transfer proteins to PVDF membranes. After blocking, the membranes were incubated with the following primary antibodies: anti‐TLR4 (1:2000), anti‐MyD88 (1:4000), anti‐NF‐κB p65 (1:1000), anti‐p‐NF‐κB p65 (1:1000), anti‐MurF1 (1:1000), anti‐MAFbx (1:1000) and anti‐GAPDH (1:30,000) overnight at 4°C. The next day, following four washes with TBST, the membranes were incubated with HRP‐conjugated anti‐Mouse IgG (1:5000) or HRP‐conjugated anti‐Rabbit IgG (1:5000) for 1 h and then washed an additional four times with TBST. Finally, immunoblots were visualized by utilizing an ECL substrate kit, and the membranes were observed and photographed using a chemiluminescence gel imaging system. The intensity of the bands was calculated using ImageJ software, with GAPDH serving as the reference standard.

### 2.15 Statistical Analysis

For statistical analysis, GraphPad Prism Software version 8.4.3 (USA) was utilized. Normality tests were performed on the data, which were reported as mean ± standard deviation (SD). A one‐way analysis of variance (ANOVA) was applied for comparisons between multiple groups. In cases of two‐group comparisons, the Student's t‐test was applied. The determination of statistical significance was based on the criterion of *p* < 0.05.

## 3 Results

### 3.1 PCA Alleviated Renal Function and PEW in CKD Rats

**FIGURE 1:**
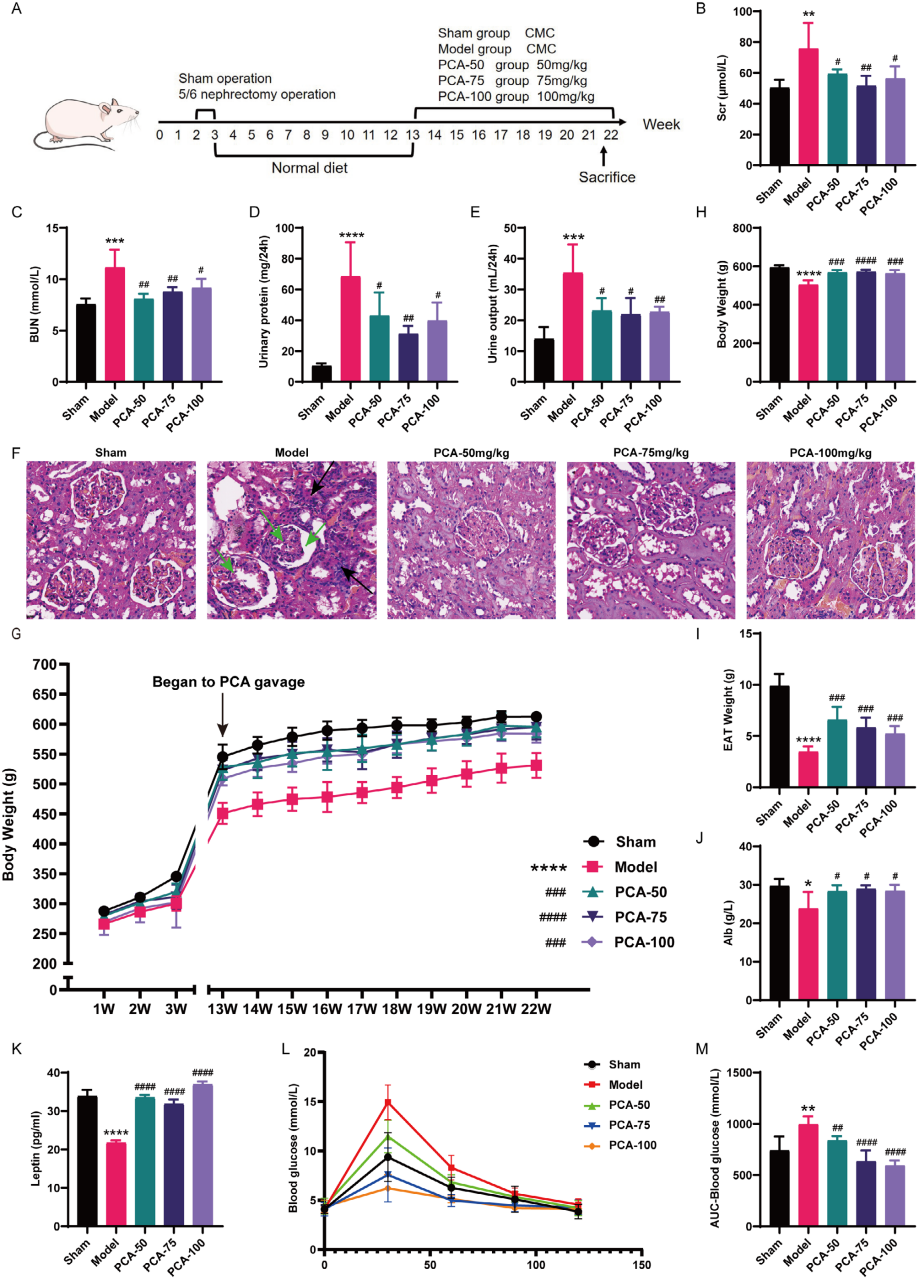
PCA alleviated renal function and protein‐energy wasting in CKD rats. (A) Animal experiment design and treatment timeline. (B–E) Scr (B), BUN (C), 24‐h urinary protein (D) and urine output (E) Levels in each group. (F) Typical photographs of H&E staining of renal tissues in each group (scale bars: 100 μm). The kidney anatomy in CKD model rats was disordered, with an extensive number of inflammatory cells infiltrating the renal interstitium (black arrow), glomeruli being significantly atrophic and cystic cavities expanding (green arrow). (G) Weekly weight fluctuations among rats in all groups. (H) Final body weight of rats on the day of sampling. (I) Weight of wet EAT in each group. (J, K) Serum Alb (J) and leptin (K) levels in each group. (L, M) Results of the IPGTT. *p < 0.05, **p < 0.01, ***p < 0.001, ****p < 0.0001, vs. the sham group; ^#^ *p* < 0.05, ^##^ *p* < 0.01, ^###^ *p* < 0.001, ^####^p < 0.0001, vs. the model group (mean ± SD, *n* = 6).

The timeline of the entire experimental procedure is illustrated in Figure 1A. The model group exhibited markedly elevated levels of Scr, BUN, 24‐h urine output and urinary protein in comparison with the sham group, whereas treatment with PCA significantly lowered these indicators of kidney injury (Figure 1B–E). H&E staining of kidney tissue revealed that the kidney anatomy in CKD model rats was disordered, with an extensive number of inflammatory cells infiltrating the renal interstitium, glomeruli being significantly atrophic and cystic cavities expanding. However, both renal inflammation and injury were alleviated after 10 weeks of PCA treatment (Figure 1F).

Besides, the results also showed the body weight status of rats belonging to various groups during the experiment (Figure 1G). The model group rats weighed considerably less than the other groups, while the PCA groups were significantly heavier (Figure 1G,H). Correspondingly, in comparison with the sham group rats, the wet weight of EAT was markedly decreased in the model group rats, while PCA treatment elevated it (Figure 1I). PCA treatment also significantly increased serum Alb and leptin levels in CKD rats compared to the model group (Figure 1J,K). The results of the IPGTT indicated that the model group had a considerably larger area under the curve (AUC) than the sham group, and this enlargement was notably diminished following PCA treatment (Figure 1L,M). In other words, PCA treatment improved insulin resistance in CKD rats.

### 3.2 PCA Alleviated Skeletal Muscle Atrophy in CKD Rats

**FIGURE 2:**
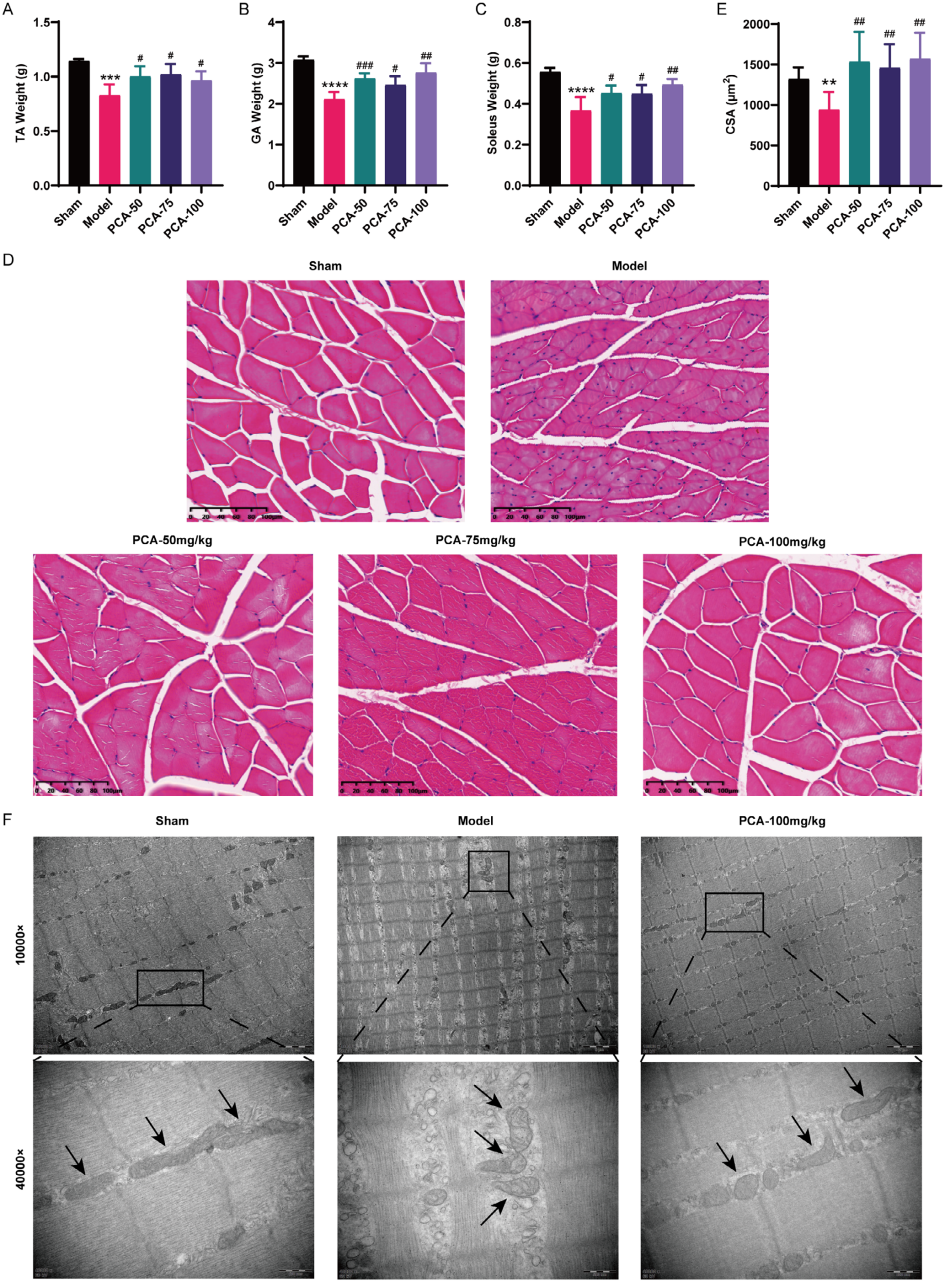
PCA alleviated skeletal muscle atrophy in CKD rats. (A–C) TA (A), GA (B) and soleus (C) Weights in each group (mean ± SD, *n* = 5–6). (D) Representative H&E‐stained images of GA (scale bars: 100 μm). (E) Muscle fibres CSA of each group (570 ~ 885 myofibres were measured in each group, mean ± SD, *n* = 6). (F) Representative electron micrographs. Mitochondria in CKD model rats were decreased, swollen, arranged disorderly and cristae were loose, accompanied by the appearance of membrane rupture or large vacuoles. The black arrows indicate mitochondria (scale bars: 2 μm and 500 nm). **p < 0.01, ***p < 0.001, ****p < 0.0001, vs. the sham group; ^#^ *p* < 0.05, ^##^ *p* < 0.01, ^###^ *p* < 0.001, vs. the model group.

In this experiment, rat body weight, muscle quantity and muscle fibre CSA were used to assess skeletal muscle atrophy. The model group exhibited markedly lower body weight, TA, GA and soleus wet weight than the sham group, while the PCA intervention group demonstrated higher values for the same parameters (Figures 1H, 2A–C). Likewise, as illustrated by H&E staining, the CSA of muscle fibres in the rat muscle tissue showed a significant decrease in the model group, and it was notably elevated in the PCA treatment group (Figure 2D,E). TEM of GA showed mitochondrial damage in the CKD model group, mainly manifested by reduced mitochondrial number and disordered arrangement, mitochondrial swelling, membrane loss, cristae loosening and vacuolization. The administration of PCA ameliorated these mitochondrial injuries in the skeletal muscle of CKD rats (Figure 2F).

### 3.3 PCA Mitigated Inflammation and Oxidative Stress in CKD Rats

**FIGURE 3:**
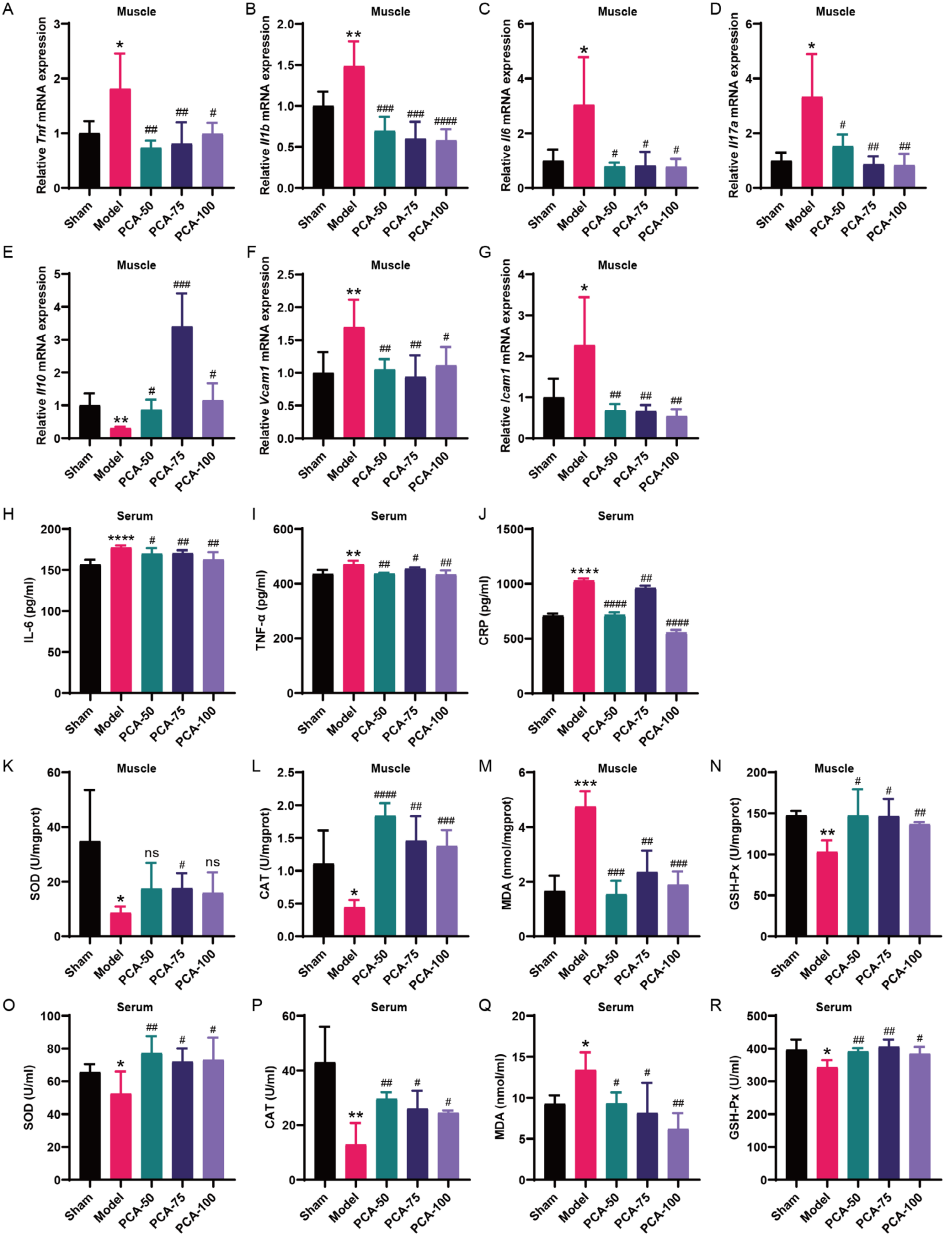
PCA mitigated inflammation and oxidative stress in CKD rats. (A–G) Levels of *Tnf, Il1b, Il6, Il17a, Il10, Vcam1* and *Icam1* inflammatory cytokines in rat muscle (mean ± SD, *n* = 4–6). (H–J) Expression of the inflammatory cytokines IL‐6, TNF‐α and CRP in serum (mean ± SD, *n* = 4–5). (K–R) Contents of SOD, CAT, MDA and GSH‐Px in serum and muscles of each group (mean ± SD, *n* = 4–6). *p < 0.05, **p < 0.01, ***p < 0.001, ****p < 0.0001, vs. the sham group; ^#^ *p* < 0.05, ^##^ *p* < 0.01, ^###^ *p* < 0.001, ^####^ *p* < 0.0001 vs. the model group.

The research findings showed that in the skeletal muscle of the model group, there was a remarkable elevation in the levels of pro‐inflammatory cytokines such as *Tnf, Il1b, Il6* and *Il17a*, accompanied by a notable suppression of the anti‐inflammatory factor *Il10*. Besides, the levels of adhesion molecules, including *Vcam1* and *Icam1* in the skeletal muscle of the model group, were also markedly elevated compared to those in the sham group. In contrast, the administration of PCA resulted in a substantial decrease in the expression of these pro‐inflammatory cytokines and adhesion molecules, along with upregulation of the anti‐inflammatory factor *Il10*, as shown in Figure 3A–G. Serum levels of the pro‐inflammatory cytokines IL‐6, TNF‐α and CRP in CKD model rats exhibited the same trend, which is also elevated in the model group and decreased in the PCA treatment group (Figure 3H–J).

In addition, relative to the sham group, the model group rats showed a marked decrease in the levels of antioxidant enzymes, including SOD, CAT and GSH‐Px, in both muscle and serum. Following PCA treatment, the activities of these enzymes were markedly elevated, nearly reaching levels comparable to those in the normal group. Additionally, the model group rats had increased levels of the peroxidation product MDA in muscle and serum, which were significantly reduced by PCA administration (Figure 3K–R).

### 3.4 Network Pharmacology Forecasted the Probable Mechanism of PCA Ameliorating Skeletal Muscle Atrophy

**FIGURE 4:**
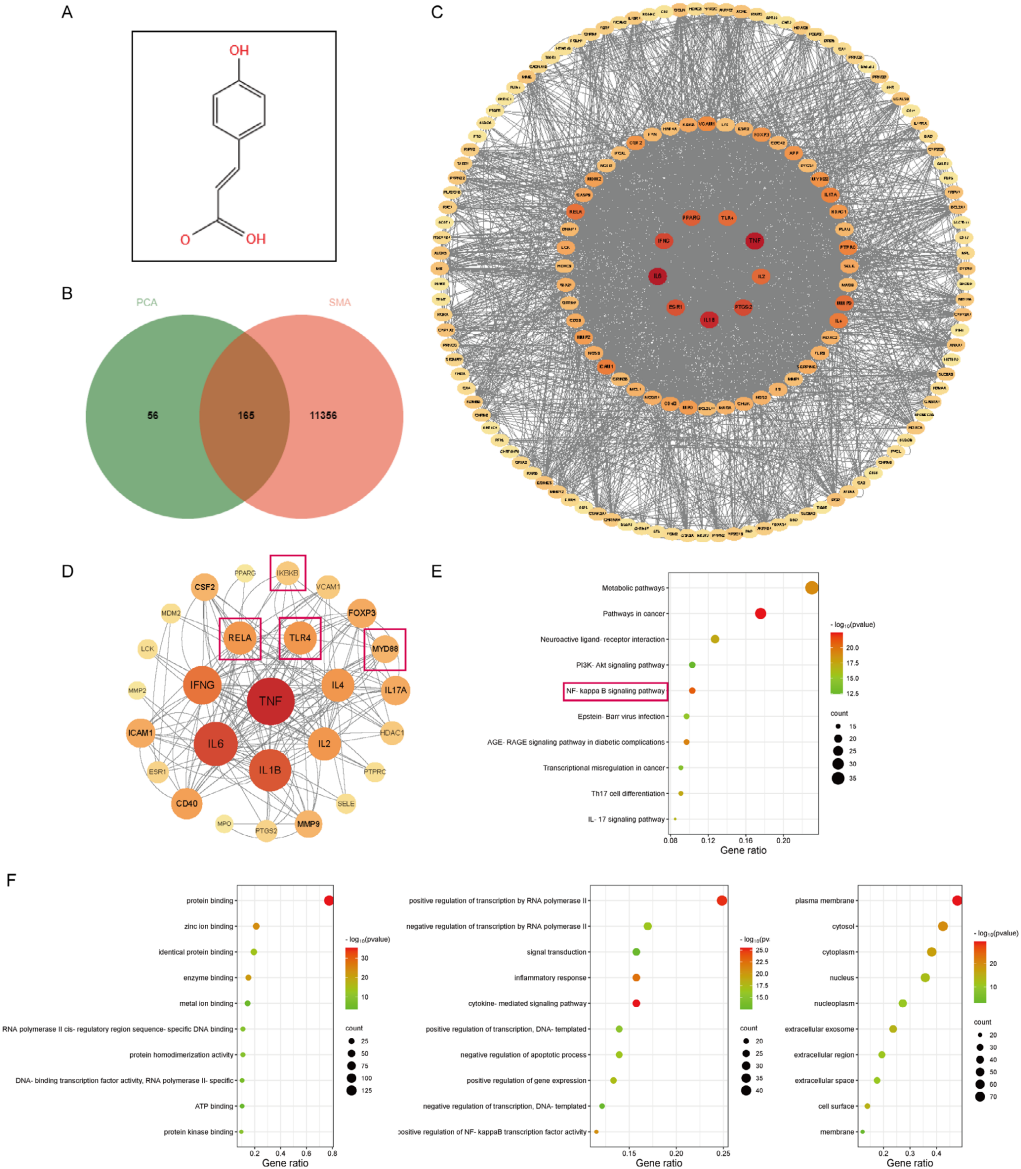
Network pharmacology forecasted the probable mechanism of PCA ameliorating skeletal muscle atrophy. (A) Molecular structure diagram of PCA. (B) Venn analysis of the intersection genes between PCA and skeletal muscle atrophy. (C) PPI network of overlapping genes. (D) PPI network of top 30 target genes. (E) KEGG enrichment analysis of overlapping genes. (F) GO analysis of overlapping genes (from left to right in turn for molecular functions, biological processes and cellular components).

For a more in‐depth exploration of the potential mechanism of PCA, a total of 221 PCA action targets and 11,521 genes related to skeletal muscle atrophy were identified, including 165 common targets between PCA and skeletal muscle atrophy (Figure 4B). The PPI network visualized with Cytoscape is shown in Figure 4C. Within this network, 164 nodes corresponded to target proteins, and 1517 edges denoted the relationships among these proteins. The intensity of the node's red colour was directly related to its degree value, signifying a robust association with the process of skeletal muscle wasting. In accordance with the degree value, the interactions among the top 30 target proteins are shown in Figure 4D. It turned out that they could interact with a large number of other proteins and occupy pivotal positions within the network, suggesting a solid foundation for subsequent investigation.

Furthermore, a total of 730 items associated with molecular functions, biological processes and cellular components were acquired from GO functional analysis. The top 30 enriched GO terms are presented in Figure 4F. These identified targets play roles in processes such as inflammation, apoptosis and signal transduction. Similarly, 151 pathways were identified through KEGG pathway enrichment analysis, and the top 10 pathways are shown in Figure 4E. These pathways primarily involve metabolism‐related pathways, tumour‐related signal pathways, the PI3K‐Akt pathway, the NF‐κB signal pathway and others.

### 3.5 Molecular Docking of PCA With Target Proteins

**FIGURE 5:**
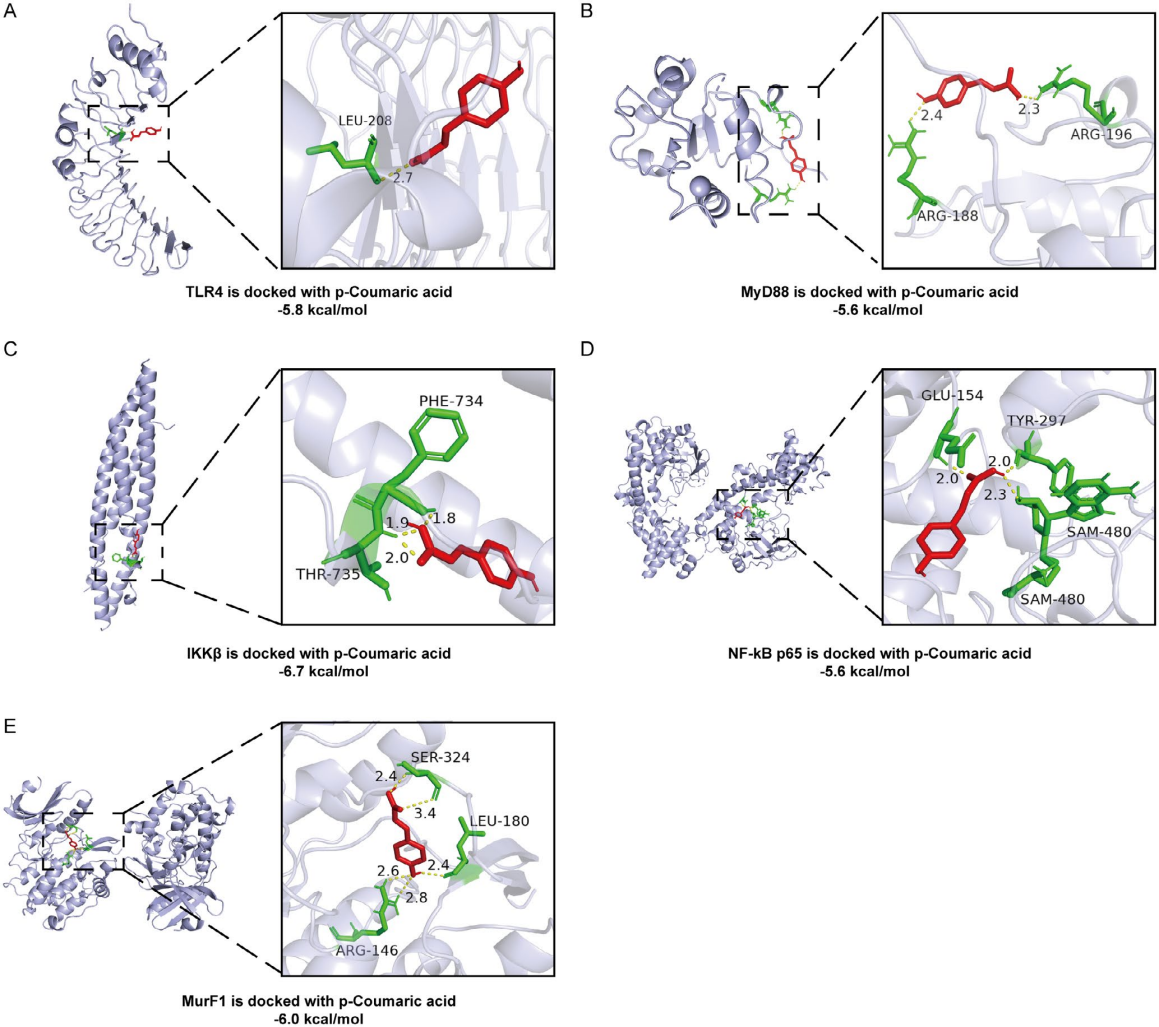
Molecular docking of PCA with target proteins. Docking situation of PCA and TLR4 (A), MyD88 (B), IKKβ (C), NF‐κB p65 (D) and MurF1 (E).

Molecular docking was performed to explore the interactions between PCA and five crucial target proteins: TLR4, MyD88, IKKβ, NF‐κB p65 and MurF1 (a marker for skeletal muscle atrophy). As presented in Table 1, the binding energies of PCA with TLR4, MyD88, IKKβ, NF‐κB p65 and MurF1 were −5.8, −5.6, −6.7, −5.6 and −6.0 kcal/mol, respectively. The molecular docking mode analysis of PCA with each target protein, shown in Figure 5A–E, revealed that PCA primarily forms stable structures with the target proteins through hydrogen bonds. Additionally, given the established role of phenolic hydroxyl groups in mediating hydroxycinnamic acids' antioxidant activity [27], the interactions of PCA and its phenolic hydroxyl‐deficient derivative CA with the same target proteins were compared to explore the correlation between the phenolic hydroxyl group and the mechanism of action of PCA. Under the condition of unified protein binding parameters or selecting the optimal binding site, the binding energy of CA to the target proteins was generally 0.2–1.6 kcal/mol lower than that of PCA (Table 1, the molecular docking mode analysis of CA with each target protein is shown in Figure S2). These results indicate that PCA exhibits strong binding affinity toward key target proteins TLR4, MyD88, IKKβ, NF‐κB p65 and MurF1, suggesting that the therapeutic effect of PCA on skeletal muscle atrophy may be mediated through the TLR4/MyD88/NF‐κB signalling pathway.

**TABLE 1:** Docking results for PCA and CA against five protein targets.

Target	Binding affinity of PCA (kcal/mol)	Binding affinity of CA (kcal/mol)
TLR4	−5.8	−5.6
MyD88	−5.6	−5.4
IKKβ	−6.7	−5.1
NF‐κB p65	−5.6	−5.2
MurF1	−6.0	−5.5

### 3.6 PCA Inhibited the TLR4/MyD88/NF‐κB Pathway in the Muscle Tissues of CKD Rats

**FIGURE 6:**
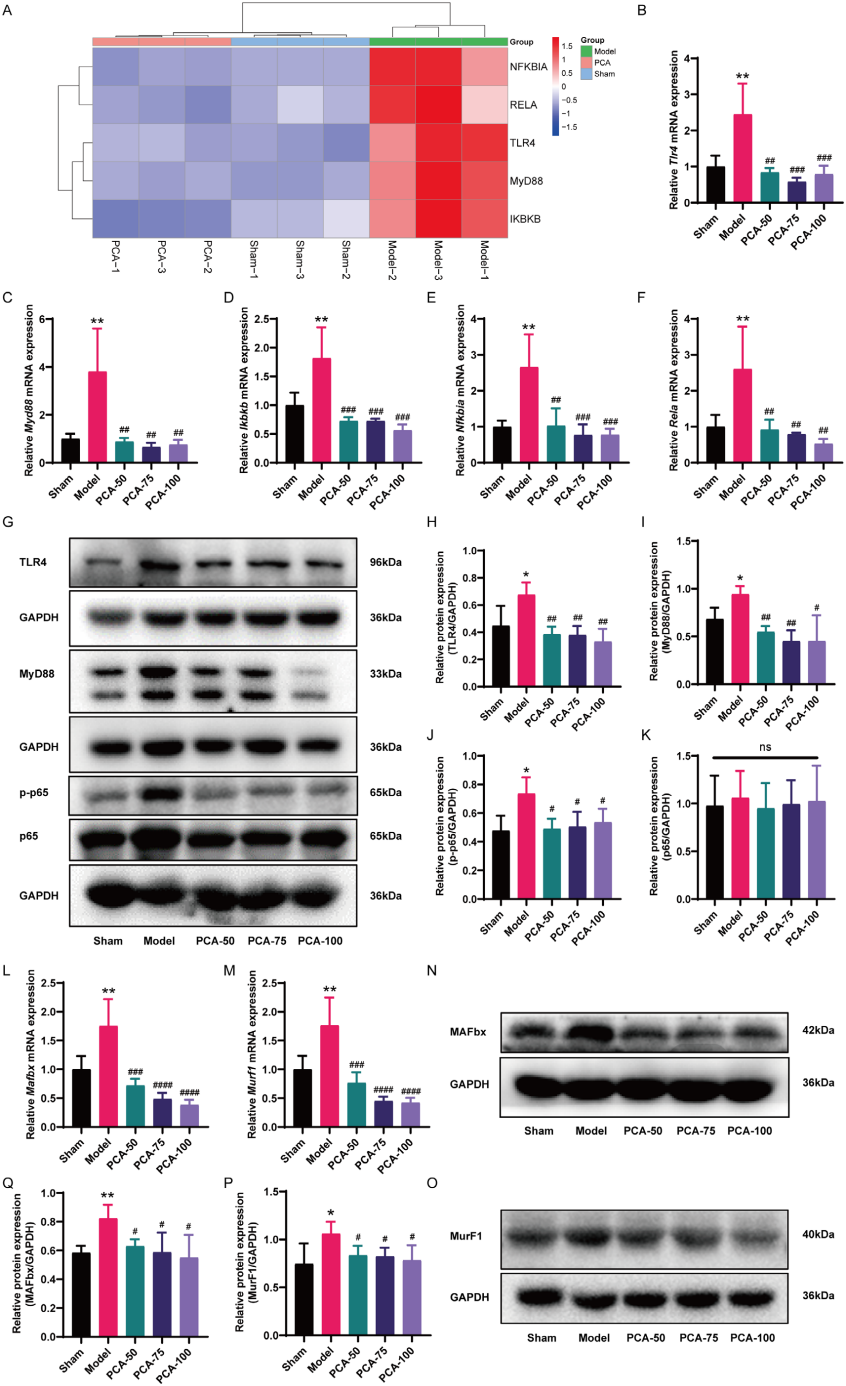
PCA inhibited the TLR4/MyD88/NF‐κB pathway in the muscle tissues of CKD rats. (A) The heatmap analysis of important genes in the TLR4/MyD88/NF‐κB signalling pathway. (B–F) Expression levels of *Tlr4* (B), *Myd88* (C), *Ikbkb* (D), *Nfkbia* (E) and *Rela* (F) in muscles of rats (mean ± SD, *n* = 6). (G) Western blot analysis of the representative bands of TLR4, MyD88, p‐p65, p65 and GAPDH proteins. (H–K) Statistical results of Western blot expression of TLR4, MyD88, p‐p65 and p65 proteins, with GAPDH as a control (mean ± SD, *n* = 3–4). (L, M) The levels of *Murf1* and *Mafbx* mRNA expression in rat muscle (mean ± SD, *n* = 6). (N, O) Representative Western blot bands of MurF1, MAFbx and GAPDH protein. (P, Q) Statistical results of Western blot expression of MurF1 and MAFbx proteins, with GAPDH as a control (mean ± SD, *n* = 4). *p < 0.05, **p < 0.01, vs. the sham group; ^#^ *p* < 0.05, ^##^ *p* < 0.01, ^###^ *p* < 0.001, ^####^ *p* < 0.0001, vs. the model group; ns, no significant differences.

In contrast to the sham rats, qRT‐PCR results revealed significantly elevated mRNA expression levels of *Tlr4, Myd88, Ikbkb, Nfkbia* and *Rela* in the skeletal muscles of CKD rats. However, PCA treatment effectively reversed these upregulations (Figure 6A–F). The Western blot results further corroborated these findings. As illustrated in Figure 6G–K, in comparison with the sham group, the skeletal muscles of CKD rats displayed markedly elevated expression levels of TLR4, MyD88 and phosphorylated p65 (p‐p65), while the total p65 protein levels showed no significant difference. Additionally, the administration of PCA effectively downregulated their expressions.

In line with the existing literature [39], the NF‐κB pathway is one of the primary triggers in the induction of atrophy stimuli and is located upstream of the proteolytic pathway mediating catabolism. Therefore, the expression of E3 ubiquitin ligases, specifically muscle‐specific ring finger 1 (MurF1) and muscle atrophy F‐box (MAFbx), in the skeletal muscles of CKD rats was examined. Our findings revealed that the mRNA expression levels of muscle atrophy markers *Murf1* and *Mafbx* were significantly elevated in the muscle of the model group, whereas these levels were notably reduced following PCA treatment (Figure 6L,M). Western blot results further indicated that PCA therapy succeeded in reversing the elevation of MurF1 and MAFbx expression in the model group (Figure 6N–Q).

### 3.7 PCA Inhibited the TLR4/MyD88/NF‐κB Signalling Pathway in C2C12 Myoblasts

**FIGURE 7:**
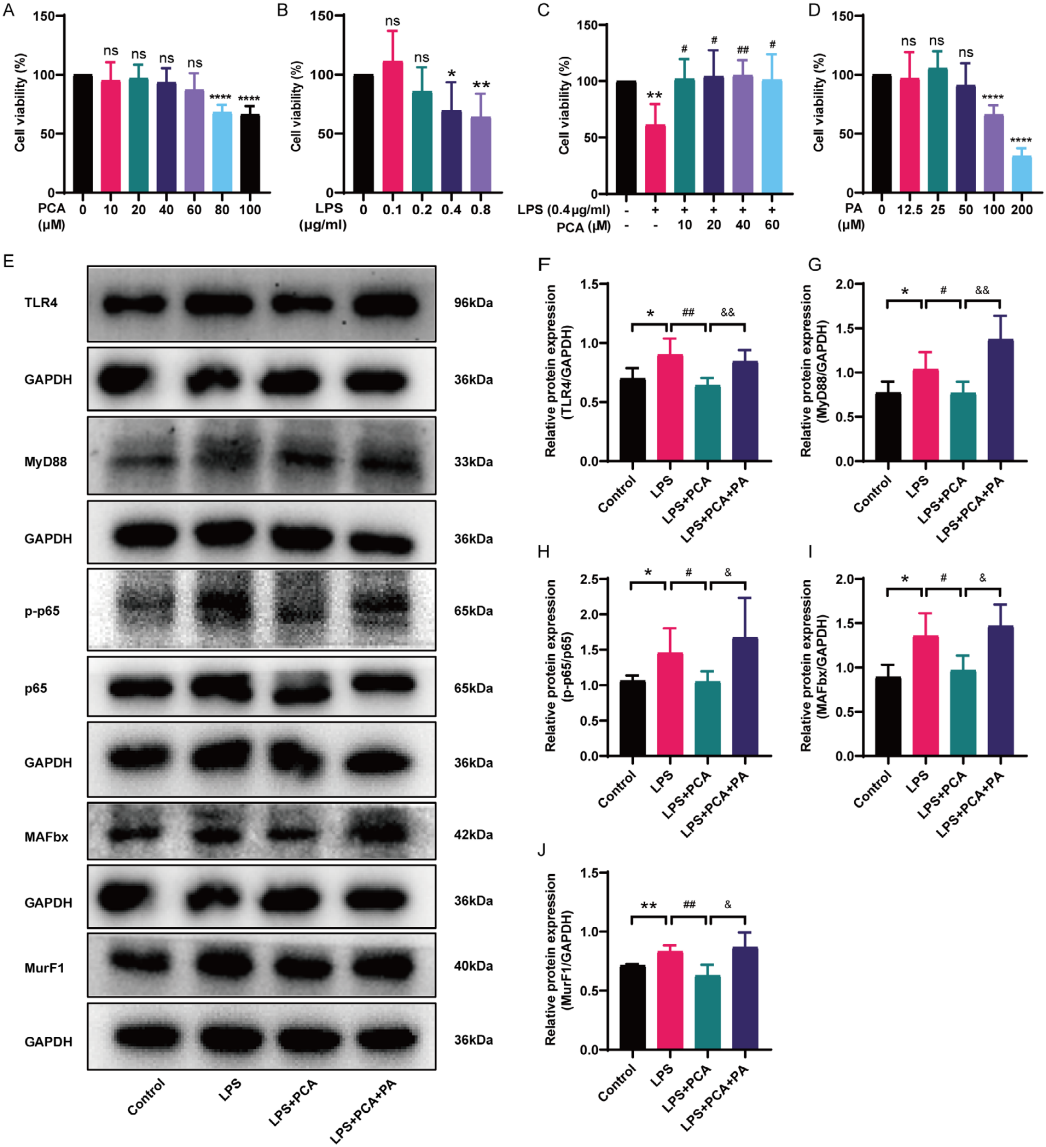
PCA inhibited the TLR4/MyD88/NF‐κB signalling pathway in C2C12 myoblasts. The MTT results showed that 40 μM PCA emerged as the most effective concentration. (A) Viability of C2C12 myoblasts after 24‐h exposure to PCA (mean ± SD, *n* = 5). (B) Viability of C2C12 myoblasts after 24‐h exposure to LPS (mean ± SD, *n* = 5). (C) LPS‐treated C2C12 myoblasts viability after treatment with PCA for 24 h (mean ± SD, *n* = 4). (D) Viability of C2C12 myoblasts after 24‐h exposure to PA (mean ± SD, *n* = 5). (E) Western blot analysis of the representative bands of TLR4, MyD88, p‐p65, p65, MAFbx, MurF1 and GAPDH proteins. (F–H) Statistical results of Western blot expression of TLR4, MyD88, p‐p65 and p65 proteins, with GAPDH as a control (mean ± SD, *n* = 5). (I, J) Statistical results of Western blot expression of MAFbx and MurF1 proteins, with GAPDH as a control (mean ± SD, *n* = 4). *p < 0.05, **p < 0.01, ****p < 0.0001, vs. the control group; ^#^ *p* < 0.05, ^##^ *p* < 0.01, vs. the LPS group; ^&^p < 0.05, ^&&^p < 0.01, vs. the LPS + PCA group.

Following the identification of PCA as a promising agent for treating CKD‐induced skeletal muscle atrophy in vivo, C2C12 myoblasts were used to substantiate the role of PCA and its underlying mechanism of action in vitro. The MTT results demonstrated that lower concentrations of PCA (10–60 μM) did not exert significant cytotoxicity, while higher concentrations (80–100 μM) produced significant cytotoxicity in C2C12 myoblasts (Figure 7A). LPS was employed to elicit inflammation and oxidative stress in C2C12 myoblasts, and a concentration of 0.4 μg/mL was selected as the effective concentration to induce significant cytotoxicity in C2C12 myoblasts (Figure 7B). PCA reversed the LPS‐induced decrease in C2C12 myoblast viability. The cell viability at 40 μM was higher than that at 10, 20 and 60 μM, so a PCA concentration of 40 μM was selected as the optimal dosage for PCA treatment (Figure 7C).

To further investigate the mechanism of action of PCA, PA, a known TLR4 agonist, was added to C2C12 myoblasts for co‐treatment with PCA. The concentration of PA at 50 μM was selected as the effective concentration due to its lack of significant cytotoxicity (Figure 7D). Western blot analysis showed that PCA suppressed the upregulation of TLR4, MyD88 and p‐p65/p65 in the TLR4/MyD88/NF‐κB signalling pathway induced by LPS in C2C12 myoblasts, ultimately leading to reduced expression levels of skeletal muscle atrophy markers MAFbx and MurF1. However, in contrast to the LPS + PCA‐treated group, the LPS + PCA + PA group exhibited elevated expression levels of TLR4, MyD88, p‐p65/p65, MAFbx and MurF1. Overall, PCA effectively suppressed the expression of proteins in the TLR4/MyD88/NF‐κB signalling pathway in C2C12 myoblasts, while the TLR4 agonist PA negated the suppressive effect of PCA (Figure 7E–J).

### 3.8 PCA Suppressed the Inflammation and Oxidative Stress Induced by LPS in C2C12 Myoblasts

**FIGURE 8:**
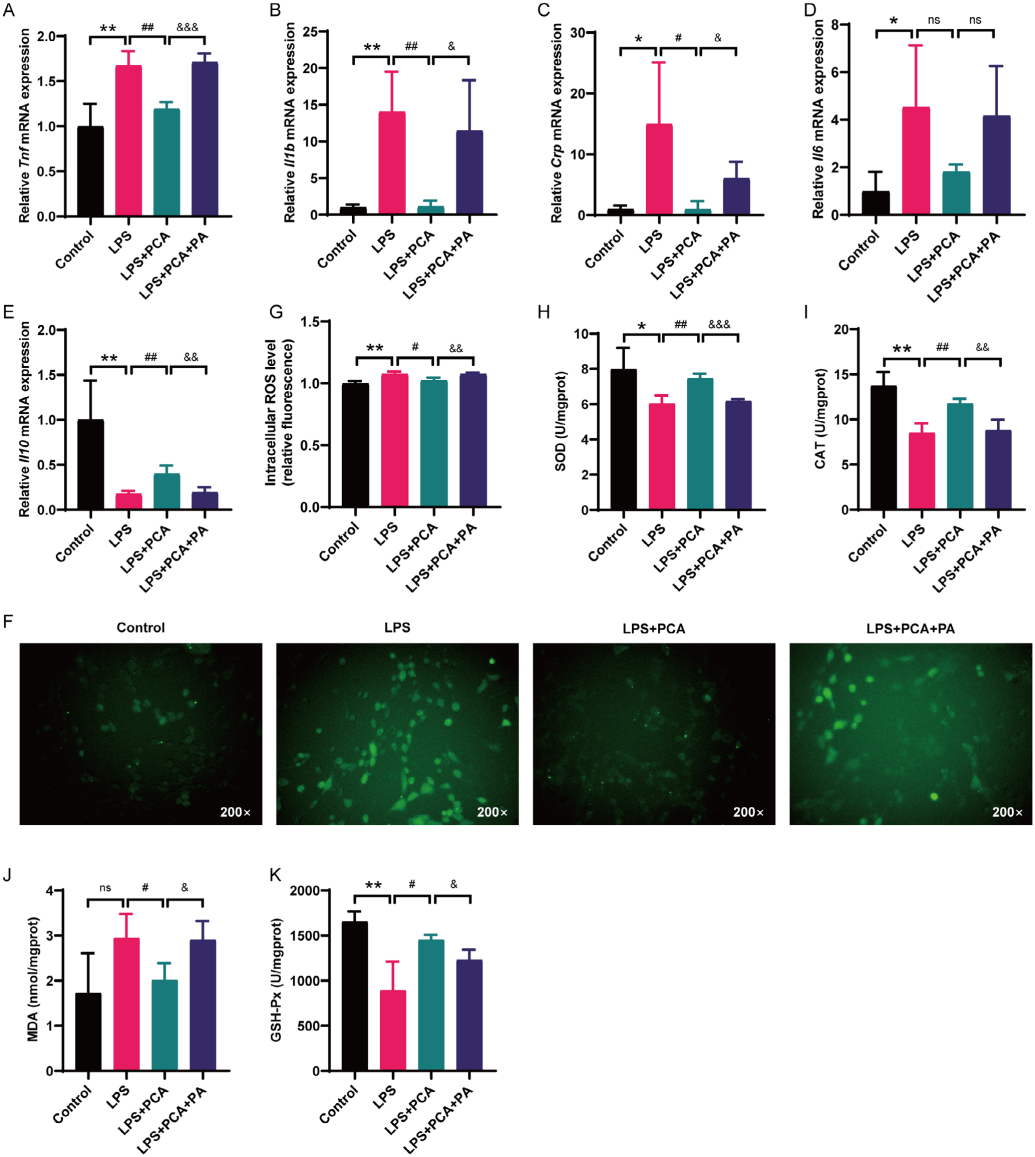
PCA suppressed the inflammation and oxidative stress induced by LPS in C2C12 myoblasts. (A–E) Expression of the inflammatory cytokines *Tnf, Il1b, Crp, Il6* and *Il10* in C2C12 myoblasts. (F) Representative pictures of ROS fluorescence in C2C12 myoblasts. (G) Figures of the statistical results of ROS fluorescence. (H–K) SOD, CAT, MDA and GSH‐Px contents in C2C12 myoblasts of different groups. *p < 0.05, **p < 0.01, vs. the control group; ^#^ *p* < 0.05, ^##^ *p* < 0.01, vs. the LPS group; ^&^ *p* < 0.05, ^&&^ *p* < 0.01, ^&&&^ *p* < 0.001, vs. the LPS + PCA group; ns, no significant difference (mean ± SD, *n* = 4).

By comparison with the LPS‐treated group, C2C12 myoblasts treated with PCA exhibited a reduction in pro‐inflammatory cytokine levels, including *Tnf, Il1b, Crp* and *Il6*, along with an increase in the concentration of the anti‐inflammatory cytokine *Il10*. Additionally, the fluorescence intensity of ROS and the content of MDA were also markedly reduced in the PCA‐treated group, accompanied by an increase in the activities of SOD, CAT and GSH‐Px. Furthermore, the co‐administration of PA counteracted the suppressive effects of PCA on LPS‐induced inflammation and oxidative stress in contrast to the group treated with PCA alone (Figure 8).

## 4 Discussion

This research detailed the beneficial effects of PCA on alleviating skeletal muscle atrophy within the context of CKD. The administration of PCA reduced levels of Scr, BUN, 24‐h urine output and urinary protein while increasing body weight, serum albumin and leptin levels, thereby ameliorating renal damage and protein‐energy malnutrition in CKD rats. PCA also increased the mass of skeletal muscles and the CSA of muscle fibres in CKD rats, thereby directly confirming its therapeutic efficacy against skeletal muscle atrophy. Concurrently, PCA suppressed the expression of pro‐inflammatory mediators in both the muscles and serum of CKD rats while enhancing the activity of antioxidant enzymes. Additionally, network pharmacology and molecular docking were performed to explore the interactions between PCA targets and genes related to skeletal muscle atrophy. Ultimately, both experiments in vivo and in vitro confirmed that PCA significantly alleviated inflammation and oxidative stress in the muscles of CKD rats and LPS‐induced C2C12 myoblasts by suppressing the TLR4/MyD88/NF‐κB signalling pathway.

The currently known mechanisms of skeletal muscle atrophy encompass oxidative stress and inflammation, the ubiquitin‐proteasome system, the autophagolysosomal system and others. These mechanisms involve numerous signalling pathways and frequently interact with each other [40, 41, 42]. In our research, PCA remarkably decreased the concentrations of pro‐inflammatory cytokines, including TNF‐α, IL‐6 and CRP, both in vivo and in vitro. Specifically, in vivo, different doses of PCA downregulated the expression of six pro‐inflammatory cytokines in the muscles of CKD rats, demonstrating distinct anti‐inflammatory effects. Inflammation is originally a self‐defence mechanism of the organism in response to infection and injury. However, in the pathological state of CKD, excessive inflammation results in the secretion of a great quantity of inflammatory cytokines, which continuously attack human muscle tissue, resulting in skeletal muscle damage or atrophy [43]. Persistent inflammation is accompanied by excessive ROS and oxidative stress [44]. The levels of ROS in the muscle tissue of CKD patients are significantly higher than those in normal individuals, with oxidative stress being a constant presence throughout the progression of muscle atrophy, from its onset to the advanced stages [45]. Our research found that PCA effectively enhanced the activities of antioxidant enzymes (SOD, CAT and GSH‐Px) and decreased the content of the peroxidation product MDA, thereby exhibiting strong antioxidant properties. Particularly in vitro, PCA effectively mitigated the accumulation of ROS in LPS‐induced C2C12 myoblasts and protected cells from oxidative damage. Interestingly, excessive ROS can not only serve as signal molecules to trigger the NF‐κB inflammatory signalling pathway and promote the persistence of inflammation [46], but also further exacerbate muscle damage by inducing lipid peroxidation and disrupting the antioxidant system [47]. Therefore, the improvement of skeletal muscle atrophy by PCA was attributed to its suppression of inflammation and oxidative stress.

After establishing the therapeutic efficacy of PCA on skeletal muscle wasting, we proceeded to delve into the underlying pathological mechanisms and cellular signalling pathways. Network pharmacology and molecular docking are two widely utilized approaches for identifying proteins, genes and pathways in drug research [48, 49]. Therefore, we employed network pharmacology to integrate the targets related to PCA and skeletal muscle atrophy, constructing a ‘PCA‐165 target‐signalling pathway’ network. Molecular docking was subsequently employed to verify the binding affinity of PCA to the identified key targets. The findings indicated that PCA exhibited strong binding interactions with TLR4, MyD88, IKKβ, NF‐κB p65 and MurF1, forming stable complexes through hydrogen bonds. These findings suggest that PCA may ameliorate CKD‐induced skeletal muscle atrophy by modulating the classical NF‐κB inflammatory signalling pathway. Notably, both network pharmacology and docking results positioned TLR4 as a significant component in PCA's mechanism of action, located upstream in the NF‐κB signalling cascade.

TLR4 is a pattern recognition receptor that recognizes pathogens and damage signals, and it is an integral part of the muscle immune response mechanism. Higher expression levels of TLR4 are observed in muscle atrophy [50, 51]. As renal function declines in CKD, uremic toxins and metabolites accumulate, acting as damage‐associated molecular patterns (DAMPs) that trigger TLR4 [52, 53]. Once activated, TLR4 recruits the adapter protein MyD88 into the receptor complex, thereby initiating the key transcription factor NF‐κB [54, 55]. NF‐κB is a nuclear transcription factor with pleiotropic regulatory effects, typically existing as a p50/p65 (RelA) heterodimer and playing a pivotal role in modulating muscle wasting [56]. Besides releasing a great quantity of pro‐inflammatory cytokines and activating the oxidative stress pathway to induce excessive ROS and oxidative stress [46], NF‐κB activation also enhances the expression of ubiquitin proteases MurF1 and MAFbx in skeletal muscle [39]. MurF1 and MAFbx, which are muscle‐specific E3 ubiquitin ligases [57], serve to identify and tag key muscle proteins, including myofibrillar proteins. These ubiquitinated proteins are then delivered to the 26S proteasome for degradation into small peptides and amino acids, thus completing the proteolytic process of muscle proteins [58, 59]. Sustained activation of NF‐κB and MurF1/MAFbx‐mediated protein degradation results in the decline of muscle mass and strength, eventually progressing to muscle wasting [60]. Therefore, inhibiting the activation of TLR4 and NF‐κB signal transduction could work jointly to combat inflammation and oxidative stress, suppress the ubiquitination process of muscle proteins and ameliorate skeletal muscle atrophy, as confirmed by our research findings. PCA suppressed the molecular activity of TLR4 and MyD88 in the skeletal muscle of CKD rats and LPS‐induced C2C12 myoblasts, thereby preventing the phosphorylation of downstream NF‐κB p65. Phosphorylation is a crucial step in the nuclear translocation of NF‐κB p65, which can amplify the transcriptional activity of NF‐κB and stimulate the expression of downstream genes [61]. The significant reduction of p‐p65 inhibited the nuclear translocation process of NF‐κB and hindered its subsequent signal transduction, thus reducing the expression levels of ubiquitin ligases MurF1 and MAFbx in the muscle of CKD rats and LPS‐induced C2C12 myoblasts, thereby inhibiting protein catabolism and ameliorating skeletal muscle wasting.

The occurrence of skeletal muscle atrophy involves numerous signalling pathways, such as NF‐κB, PI3K/Akt and TGF‐β/Myostatin [40]. Our study primarily attributed PCA's mechanism of action to its regulation of the TLR4‐mediated NF‐κB signalling pathway rather than other pathways, due to its inhibitory effects on skeletal muscle inflammation and oxidative stress, as well as its direct suppression of the TLR4/MyD88/NF‐κB signalling pathway. In addition, in vitro experiments, the combined use of PA, a common TLR4 agonist [62], further substantiated this conclusion. The results showed that the co‐treatment with PA partially eliminated the suppressive effects of PCA on TLR4, MyD88 and the NF‐κB signalling transduction pathway in LPS‐induced C2C12 myoblasts, including the inhibitory effects of PCA on E3 ubiquitin ligases MurF1 and MAFbx. Similarly, the suppressive effects of PCA on LPS‐induced inflammation and oxidative stress in C2C12 myoblasts were also reversed by PA. This further elucidated that the beneficial regulatory effects of PCA on inflammation, oxidative stress and the ubiquitination system in muscle cells were, at least in part, mediated by the inhibition of the TLR4‐mediated NF‐κB signalling pathway. The inhibition of TLR4 by PCA partially blocked the activation of NF‐κB. This impeded NF‐κB transduction, suppressed the production of pro‐inflammatory cytokines and ROS, induced the expression of various antioxidant enzymes and also reduced the expression levels of ubiquitin ligases, inhibiting protein degradation, thus exerting a therapeutic effect on skeletal muscle atrophy. These insights underscore the therapeutic potential of PCA as a natural compound for the improvement of skeletal muscle atrophy in CKD. However, the pharmacological actions of PCA are extensive, yet the in‐depth structure–activity relationship between these actions and the phenolic hydroxyl group structure remains to be further investigated.

## 5 Conclusion

**FIGURE 9:**
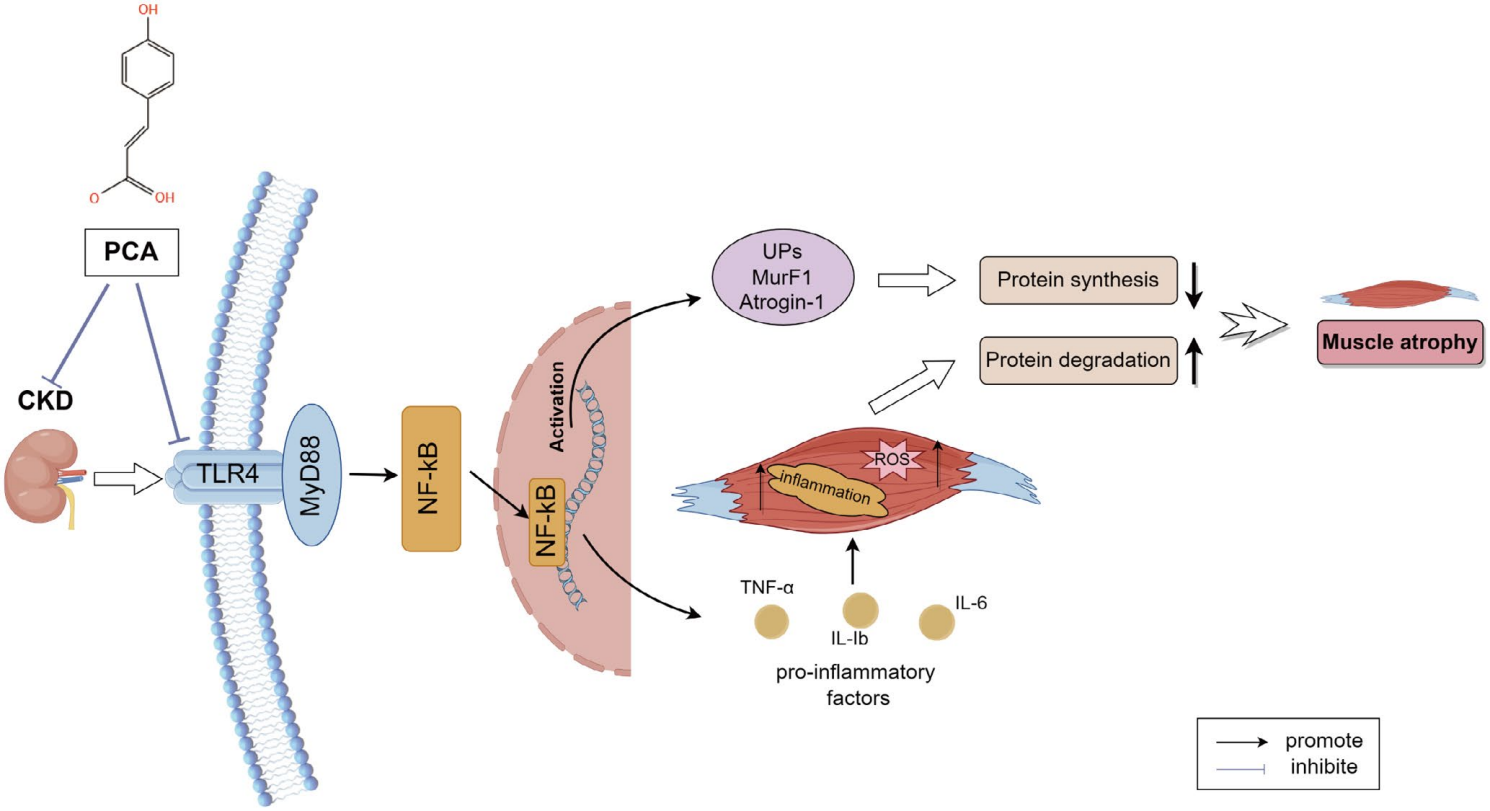
A brief overview of PCA's mechanism for alleviating skeletal muscle atrophy in CKD (by Figdraw).

Our study proposes that PCA exerts its anti‐inflammatory and antioxidant effects to ameliorate skeletal muscle atrophy through the inhibition of the TLR4/MyD88/NF‐κB signalling pathway. The mechanism of action of PCA is illustrated in Figure 9.

## Author Contributions

**Hao Wang:** methodology (equal), validation (equal), formal analysis (equal) and writing – original draft (equal); **Chi Zhang:** conceptualization (equal), data curation (equal) and formal analysis (equal); **Jinyue He:** validation (equal), software (equal) and investigation (equal); **Zhuoen He** and **Shihua Yan:** software (equal) and formal analysis (equal); **Yuan Zhang** and **Shiyin Huang:** software (equal) and investigation (equal); **Yangtian Yan**, **Yuchi Chen** and **Zhiqiang Xian:** investigation (equal); **Rong Hu:** funding acquisition (equal); **Yanjing Wang:** resources (equal); **Wei Xiao** and **Mingqing Wang:** project administration (equal), resources (equal), writing – review and editing (equal), supervision (equal) and funding acquisition (equal).

## Ethics Statement

All animal experimental studies were subject to review and approval by the Institutional Animal Care and Use Committee of Southern Medical University (Grant No.: 2022056) and were conducted following ethical principles.

## Conflicts of Interest

The authors declare no conflicts of interest.

## Supporting information


**Figure S2.** Molecular docking of CA with target proteins.

**Table S1.** Primer sequences used for qRT‐PCR.

## Data Availability

The data that support the findings of this study are available from the corresponding author upon reasonable request. More data that support the findings of this study are available in Table S1 and Figure S2 of this article.
