# Supplementary figures and images for: P‐Coumaric Acid Improves Skeletal Muscle Atrophy in Chronic Kidney Disease by Modulating TLR4/MyD88/NF‐κB‐Mediated Inflammation and Oxidative Stress
Source: J Cell Mol Med. 2025 Jul 17;29(14):e70659. doi: 10.1111/jcmm.70659 (PMC12268968; doi:10.1111/jcmm.70659)

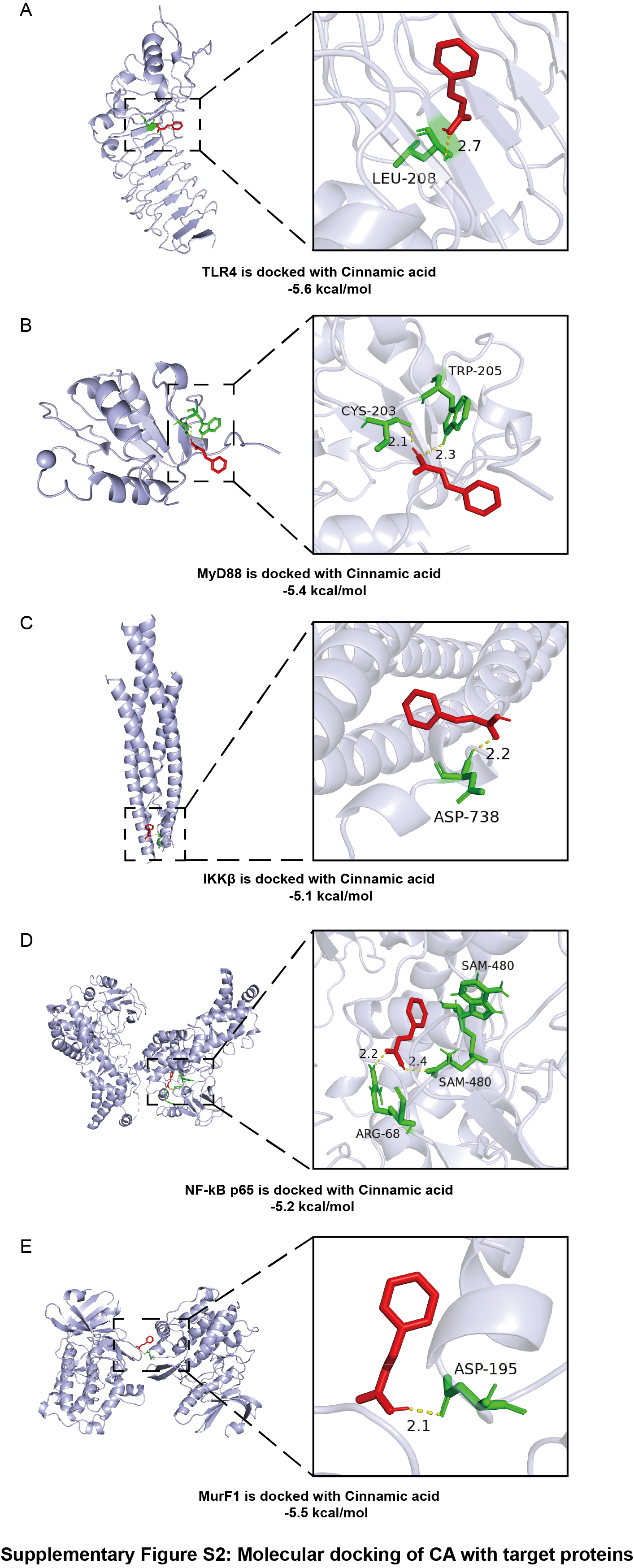

Supplement: Supplementary file 1 — Figure S2. Molecular docking of CA with target proteins. [file JCMM-29-e70659-s001.tif]
